# Evaluation about the Performance of E-Government Based on Interval-Valued Intuitionistic Fuzzy Set

**DOI:** 10.1155/2014/234241

**Published:** 2014-02-23

**Authors:** Shuai Zhang, Dejian Yu, Yan Wang, Wenyu Zhang

**Affiliations:** School of Information, Zhejiang University of Finance and Economics, Hangzhou 310018, China

## Abstract

The evaluation is an important approach to promote the development of the E-Government. Since the rapid development of E-Government in the world, the E-Government performance evaluation has become a hot issue in the academia. In this paper, we develop a new evaluation method for the development of the E-Government based on the interval-valued intuitionistic fuzzy set which is a powerful technique in expressing the uncertainty of the real situation. First, we extend the geometric Heronian mean (GHM) operator to interval-valued intuitionistic fuzzy environment and proposed the interval-valued intuitionistic fuzzy GHM (IIFGHM) operator. Then, we investigate the relationships between the IIFGHM operator and some existing ones, such as generalized interval-valued intuitionistic fuzzy HM (GIIFHM) and interval-valued intuitionistic fuzzy weighted Bonferoni mean operator. Furthermore, we validate the effectiveness of the proposed method using a real case about the E-Government evaluation in Hangzhou City, China.

## 1. Introduction

Intuitionistic fuzzy set (IFS), an extension of Zadeh's fuzzy set, was first proposed by Atanassov [1]. Over the last decade, the IFS theory issue has become an important research area of mathematics, management, and computer sciences. It is generally known that the membership degree and nonmembership degree of the IFS are expressed by determined number [2–10]. Based on the IFS theory, Atanassov and Gargov [11] utilized the interval number rather than the determined number to express the membership degree and nonmembership degree and introduced the interval-valued IFS (IIFS). Researchers have many research works and have some results regarding IIFS theory.

Interval-valued intuitionistic fuzzy number (IIFN) is the basic ingredient of the IIFS theory and more powerful to express the uncertainty than intuitionistic fuzzy number (IFN) [12–14]. How to aggregate the IIFNs to a comprehensive one is a very active research area and is critical for artificial intelligence, decision making, and management science. So far there are many aggregation operators proposed to aggregate the IIFNs [15–17]. The Heronian mean (HM) is a mean type information aggregation technique, which is proposed by Beliakov et al. [18] and mainly used to aggregate determined numbers. In this paper, we extend the HM mean operator to adapt it to interval-valued intuitionistic fuzzy environment and then study the E-Government evaluation method based on IIFS theory.

To do this, we organize the paper as follows.  Section 2 extends the GHM operator to interval-valued intuitionistic fuzzy environment and proposes the interval-valued intuitionistic fuzzy GHM (IIFGHM) operator. Some special cases are discussed in this section.  Section 3 introduces the interval-valued intuitionistic fuzzy geometric weighted Heronian mean (IIFGWHM) and develops an approach for multicriteria decision making. A real case about the E-Government evaluation in Hangzhou City, China, is also provided in this section.  Section 4 ends this paper with some concluding remarks.

## 2. The Interval-Valued Intuitionistic Fuzzy Geometric Heronian Mean Operator

Atanassov and Gargov [11] first proposed the IIFS and gave the definition of IIFS.

**Definition 1**

The IIFS *A* on *X* was defined as follows:

(1) $$\begin{array}{c} A=\left\{\langle x,{\tilde{t}}_{A}\left(x\right),{\tilde{f}}_{A}\left(x\right)\rangle \;|\;x\in X\right\}. \end{array}$$

The ${\tilde{t}}_{A}\left(x\right)$ and ${\tilde{f}}_{A}\left(x\right)$ are two functions that indicated the degrees range of membership and nonmembership, respectively. Furthermore, the two functions are valued between [0, 1] and the sum of the maximum value of the two functions is also between [0, 1] [19].

**Definition 2 (see [20])**

Let ${\tilde{\alpha }}_{1}=([a_{1},b_{1}],[c_{1},d_{1}])$ and ${\tilde{\alpha }}_{2}=([a_{2},b_{2}],[c_{2},d_{2}])$ be any two IIFNs; then some operational rules of IIFN ${\tilde{\alpha }}_{1}$ and IIFN ${\tilde{\alpha }}_{2}$ are defined as

1. ${\tilde{\alpha }}_{1}⊕{\tilde{\alpha }}_{2}=(\left[a_{1}+a_{2}-a_{1}a_{2},b_{1}+b_{2}-b_{1}b_{2}\right],[c_{1}c_{2},d_{1}d_{2}])$;
2. ${\tilde{\alpha }}_{1}⊗{\tilde{\alpha }}_{2}=(\left[a_{1}a_{2},b_{1}b_{2}\right],[c_{1}+c_{2}-c_{1}c_{2},d_{1}+d_{2}-d_{1}d_{2}])$;
3. $\lambda {\tilde{\alpha }}_{1}=([1-{\left(1-a_{1}\right)}^{\lambda },1-{\left(1-b_{1}\right)}^{\lambda }],[c_{1}^{\lambda },d_{1}^{\lambda }]),\;\;\lambda >0$;
4. ${{\tilde{\alpha }}_{1}}^{\lambda }=([a_{1}^{\lambda },b_{1}^{\lambda }],[1-{\left(1-c_{1}\right)}^{\lambda },1-{\left(1-d_{1}\right)}^{\lambda }]),\;\;\lambda >0$.

And the score function of IIFN ${\tilde{\alpha }}_{1}$ is defined as

(2) $$\begin{array}{c} s\left({\tilde{\alpha }}_{1}\right)=\frac{a_{1}+b_{1}-c_{1}-d_{1}}{2}. \end{array}$$

The score function of IIFN is an important indicator for comparing any two IIFNs. In the general case, the bigger the score function, the bigger the IIFN.

**Example 3**

Let ${\tilde{\alpha }}_{1}=([0.5,0.6],[0.2,0.3])$, ${\tilde{\alpha }}_{2}=([0.1,0.3],[0.4,0.6])$, and ${\tilde{\alpha }}_{3}=([0.3,0.6],[0.3,0.2])$ be three IIFNs; we can get the following score functions based on (2)

(3) $$\begin{array}{c} s\left({\tilde{\alpha }}_{1}\right)=\frac{0.5+0.6-0.2-0.3}{2}=0.3,\\ s\left({\tilde{\alpha }}_{2}\right)=\frac{0.1+0.3-0.4-0.6}{2}=-0.3,\\ s\left({\tilde{\alpha }}_{3}\right)=\frac{0.3+0.6-0.3-0.2}{2}=0.2. \end{array}$$

Since

(4) $$\begin{array}{c} s\left({\tilde{\alpha }}_{1}\right)>s\left({\tilde{\alpha }}_{3}\right)>s\left({\tilde{\alpha }}_{2}\right), \end{array}$$

then

(5) $$\begin{array}{c} {\tilde{\alpha }}_{1}≻{\tilde{\alpha }}_{3}≻{\tilde{\alpha }}_{2}. \end{array}$$

Heronian mean (HM) is able to characterize quantitatively the relations between the aggregated arguments. The definition of HM was given as follows.

**Definition 4 (see [18])**

Let *a*_*i*_  (*i* = 1,2,…, *n*) be a collection of nonnegative numbers. If

(6) $$\begin{array}{c} \text{HM}\left(a_{1},a_{2},\ldots ,a_{n}\right)=\frac{2}{n\left(n+1\right)}\overset{n}{\underset{i=1}{\sum }}\overset{n}{\underset{j=i}{\sum }}\sqrt{a_{i}a_{j}}, \end{array}$$

then HM is called the Heronian mean (HM).

**Example 5**

Let *a*_1_ = 2, *a*_2_ = 3, *a*_3_ = 4 be three nonnegative numbers; based on the HM operator, we can get

(7) HM(a1,a2,a3) =23(3+1)(2×2+2×3+2×4+3×3+3×4+4×4)=2.96.

Based on Definition 4, Yu [21] proposed the geometric Heronian mean as follows.

**Definition 6**

Let *p* > 0, *q* > 0, *a*_*i*_  (*i* = 1,2,…, *n*) be a collection of nonnegative numbers. If

(8) $$\begin{array}{c} \text{GHM}\left(a_{1},a_{2},\ldots ,a_{n}\right)=\frac{1}{p+q}\left(\overset{n}{\underset{i=1,j=i}{\prod }}{\left(pa_{i}+qa_{j}\right)}^{2/n(n+1)}\right), \end{array}$$

then GHM is called the geometric Heronian mean (GHM).

In order to deal with the situation of interval-valued intuitionistic fuzzy environment, we extend the GHM and propose the interval-valued intuitionistic fuzzy GHM as follows.

**Definition 7**

Let *p* > 0, *q* > 0, ${\tilde{\alpha }}_{i}=\left(\left[a_{i},b_{i}\right],\left[c_{i},d_{i}\right]\right)\;\;(i=1,2,\ldots ,n)$ be a collection of IIFNs; if

(9) IIFGHM(α~1,α~2,…,α~n) =1p+q(⨂i=1,j=in(pα~i+qα~j)2/n(n+1)),

then IIFGHM is called the interval-valued intuitionistic fuzzy geometric Heronian mean (IIFGHM).

Based on the operational laws of the IIFNs described in Definition 2, we can derive the following results.

**Theorem 8**

Let *p* > 0, *q* > 0, ${\tilde{\alpha }}_{i}=\left(\left[{\text{a}}_{i},b_{i}\right],\left[c_{i},d_{i}\right]\right)\;\;(i=1,2,\ldots ,n)$ be a collection of IIFNs; then the aggregated value by using the IIFGHM is also an IIFN, and

(10) IIFGHM(α~1,α~2,…,α~n) =([1−(1−∏i=1,j=in(1−(1−ai)p×(1−aj)q)2/n(n+1))1/(p+q), 1−(1−∏i=1,j=in(1−(1−bi)p×(1−bj)q)2/n(n+1))1/(p+q)], [(1−∏i=1,j=in(1−cipcjq)2/n(n+1))1/(p+q),(1−∏i=1,j=in(1−dipdjq)2/n(n+1))1/(p+q)]).

**Proof**

We can prove Theorem 8 by mathematical induction and the similar proof method can be referred to Yu [21].

We studied the interval-valued intuitionistic fuzzy Heronian mean and proposed the generalized interval-valued intuitionistic fuzzy HM (GIIFHM) in our previous works [22]. It should be noted that the GIIFHM operator is a kind of averaging mean operator and the IIFGHM proposed in this paper is a kind of geometric mean operator. We try to apply a numeric example in simulation in order to compare the IIFGHM and GIIFHM operators.

**Example 9**

**Figure 1:**
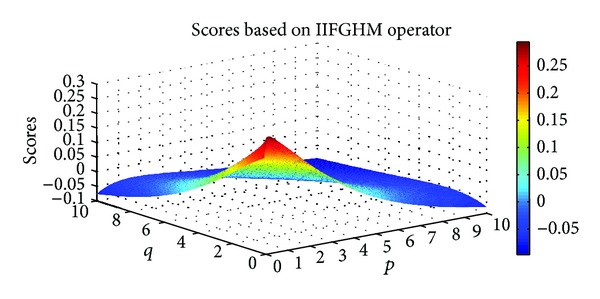
Scores obtained by the IIFGHM operator (*p* ∈ (0,10], *q* ∈ (0,10]).

**Figure 2:**
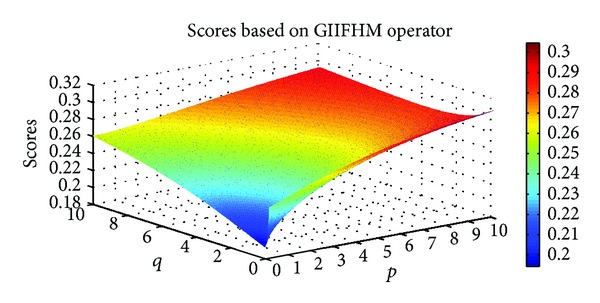
Scores obtained by the GIIFHM operator (*p* ∈ (0,10], *q* ∈ (0,10]).

Let ${\tilde{\alpha }}_{1}=([0.7,0.8],[0.1,0.2])$, ${\tilde{\alpha }}_{2}=([0.3,0.4],[0.5,0.6])$, and ${\tilde{\alpha }}_{3}=([0.6,0.7],[0.2,0.3])$ be three IIFNs; when the parameters *p*, *q* take different values, scores values are obtained based on IIFGHM and GIIFHM operators which are shown in Figures 1 and 2.

## 3. Interval-Valued Intuitionistic Fuzzy Multicriteria Decision Making Based on IIFGWHM Operator

The IIFGHM operator does not consider the weight of the aggregated arguments and it should be improved. In this section we first introduce the weighted form of IIFGHM (IIFGWHM) operator and then introduce a multicriteria decision making method based on IIFGWHM operator.

**Definition 10**

Let ${\tilde{\alpha }}_{i}=\left(\left[a_{i},b_{i}\right],\left[c_{i},d_{i}\right]\right)\;\;(i=1,2,\ldots ,n)$ be a collection of IIFNs and *w* = (*w*_1_, *w*_2_,…, *w*_*n*_)^*T*^ be the weight vector of ${\tilde{\alpha }}_{i}\;\;(i=1,2,\ldots ,n)$, where *w*_*i*_ indicates the importance degree of *α*_*i*_, satisfying *w*_*i*_ > 0, *i* = 1,2,…, *n*, and ∑_*i*=1_^*n*^ *w*_*i*_ = 1. If

(11) IIFGWHMp,q(α~1,α~2,…,α~n) =1p+q(⨂i=1,j=in((pα~i)wi⊕(qα~j)wj)2/n(n+1)),

then IIFGWHM is called the interval-valued intuitionistic fuzzy geometric weighted Heronian mean (IIFGWHM).

Similar to Theorem 8, Theorem 11 can be derived easily.

**Theorem 11**

Let *p* > 0, *q* > 0, ${\tilde{\alpha }}_{i}=\left(\left[a_{i},b_{i}\right],\left[c_{i},d_{i}\right]\right)\;\;(i=1,2,\ldots ,n)$ be a collection of IIFNs, whose weight vector is *w* = (*w*_1_, *w*_2_,…, *w*_*n*_)^*T*^, which satisfies *w*_*i*_ > 0, *i* = 1,2,…, *n*, and ∑_*i*=1_^*n*^ *w*_*i*_ = 1. Then the aggregated value by using the IIFGWHM is also an IIFN, and

(12) IIFGWHM(α~1,α~2,…,α~n) =([1−(1−∏i=1,j=in(1−(1−aiwi)p×(1−ajwj)q)2/n(n+1))1/(p+q), 1−(1−∏i=1,j=in(1−(1−biwi)p×(1−bjwj)q)2/n(n+1))1/(p+q)], [(1−∏i=1,j=in(1−(1−(1−cj)wi)p×(1−(1−ci)wj)q)2/n(n+1))1/(p+q), (1−∏i=1,j=in(1−(1−(1−di)wi)p×(1−(1−dj)wj)q)2/n(n+1))1/(p+q)]).

In a presumed multicriteria decision making problem [23–29], let *A* = {*A*_1_, *A*_2_,…, *A*_*m*_} be a set of *m* Districts and let *C* = {*c*_1_, *c*_2_,…, *c*_*n*_} a set of *n* criteria, whose weight vector is *w* = (*w*_1_, *w*_2_,…, *w*_*n*_)^*T*^, satisfying *w*_*j*_ > 0, *j* = 1,2,…, *n* and ∑_*j*=1_^*n*^ *w*_*j*_ = 1. The performance of District *A*_*i*_ with respect to the criterion *c*_*j*_ is measured by an IIFN ${\tilde{\alpha }}_{ij}=\left(\left[a_{ij},b_{ij}\right],\left[c_{ij},d_{ij}\right]\right)\;\;(i=1,2,\ldots ,m;\;\;j=1,2,\ldots ,n)$, where [*a*_*ij*_, *b*_*ij*_] indicates the degree range in which District *A*_*i*_ satisfies the criterion *c*_*j*_ and [*c*_*ij*_, *d*_*ij*_] indicates the degree range in which District *A*_*i*_ does not satisfy the criterion *c*_*j*_ and construct the interval-valued intuitionistic fuzzy decision matrix $D={\left({\tilde{\alpha }}_{ij}\right)}_{n\times m}$.

**Step 1**

Normalize the decision making matrix into standardized matrix. In other words, if the criteria *c*_*j*_ is the benefit criteria, then the values do not need changing; if criteria *c*_*j*_ is the cost criteria, then use ${\overset{-}{\tilde{\alpha }}}_{j}$ instead of ${\tilde{\alpha }}_{j}$ [30–33], where ${\overset{-}{\tilde{\alpha }}}_{j}=\left(\left[c_{j},d_{j}\right],\left[a_{j},b_{j}\right]\right)$ is the complement of ${\tilde{\alpha }}_{j}=\left(\left[a_{j},b_{j}\right],\left[c_{j},d_{j}\right]\right)$.

**Step 2**

Aggregate all the performance values ${\tilde{\alpha }}_{ij}\;\;(j=1,2,\ldots ,n)$ of the *i*th line, and get the overall performance value ${\tilde{\alpha }}_{i}$ corresponding to District *A*_*i*_ by the IIFGWHM:

(13) IIFGWHMp,q(α~1,α~2,…,α~n) =1p+q(⨂i=1,j=in((pα~i)wi⊕(qα~j)wj)2/n(n+1)),

where *p*, *q* > 0.

**Step 3**

Rank the overall performance values ${\tilde{\alpha }}_{i}\;\;(i=1,2,\ldots ,m)$ according to Definition 2 and obtain the priority of Districts *A*_*i*_  (*i* = 1,2,…, *m*) according to ${\tilde{\alpha }}_{i}\;\;(i=1,2,\ldots ,m)$.

**Example 12**

Advocating the E-Government has important value for establishing a harmonious and efficient government. Experience has confirmed the potential effect of E-Government on the development of whole society. It is a fact to the academic circles that the continual development of E-Government needs the support of the performance evaluation. Hangzhou city is the capital of Zhejiang Province, China, and is the political, economic, cultural, and financial and transportation center of Zhejiang Province. At present, the performances of the E-Government of the four Districts *A*_*i*_  (*i* = 1,2,…, 4) in Hangzhou city need to be evaluated. Based on the result of many researches [34, 35], this evaluation proceeds in the following three aspects: construction costs of the E-Government (*C*_1_), the effectiveness of the E-Government system (*C*_2_), and the quality of E-Government system (*C*_3_). The three criteria may occur to different degrees and suppose (0.27,0.46,0.27)^*T*^ as the weight vector of the three criteria. The evaluation information on the four Districts *A*_*i*_  (*i* = 1,2,…, 4) under the factors *C* = {*c*_1_, *c*_2_, *c*_3_} are represented by the IIFNs and shown in Table 1.

Since the construction costs of the E-Government (*C*_1_) is the cost criteria, therefore, it needs normalization. The Normalized decision matrix is shown in Table 2.

From the Definition of IIFGWHM operator, we know that the values of parameters *p* and *q* may largely affect the aggregated IIFEs. In the following, we study the aggregated results as the values of the parameters *p* and *q* change. Tables 3 and 4 show the details of the results.

**Figure 3:**
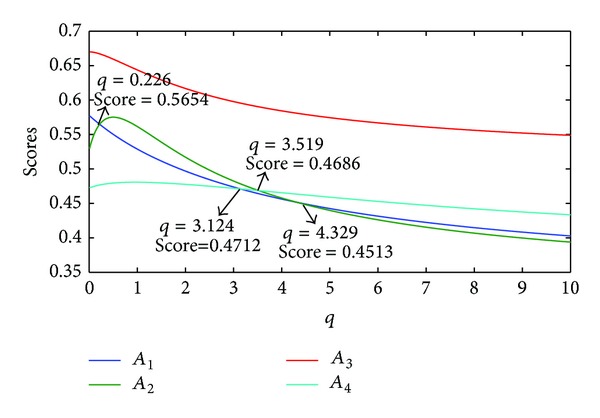
Scores of IIFWGHM (*p* = 1, *q* ∈ (0,10]).

If we let the parameter *p*  (*p* = 1) be fixed, different scores and rankings of the Districts can be obtained as the parameter *q* changes, as is shown in Figure 3.

From Figure 3, we can find that,

1. when *q* ∈ (0,0.226], the ranking order of the Districts is *A*_3_≻*A*_1_≻*A*_2_≻*A*_4_,
2. when *q* ∈ [0.226,3.124], the ranking order of the Districts is *A*_3_≻*A*_2_≻*A*_1_≻*A*_4_,
3. when *q* ∈ [3.124,3.519], the ranking order of the Districts is *A*_3_≻*A*_2_≻*A*_4_≻*A*_1_,
4. when *q* ∈ [3.519,4.329], the ranking order of the Districts is *A*_3_≻*A*_4_≻*A*_2_≻*A*_1_,
5. when *q* ∈ [4.329,10.0], the ranking order of the Districts is *A*_3_≻*A*_4_≻*A*_1_≻*A*_2_.

**Figure 4:**
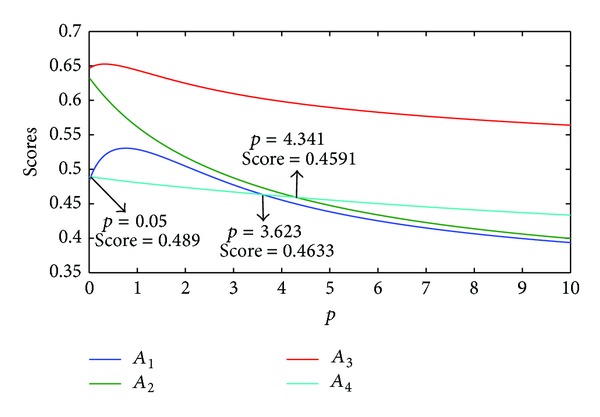
Scores of IIFGWHM (*q* = 1, *p* ∈ (0,10]).

On the other hand, if we let the parameter *q*  (*q* = 1) be fixed, different rankings of the Districts can be obtained as the parameter *p* changed which was shown in Figure 4.

From Figure 4, we can find that,

1. when *q* ∈ (0,0.05], the ranking order of the Districts is *A*_3_≻*A*_2_≻*A*_4_≻*A*_1_,
2. when *q* ∈ [0.05,3.623], the ranking order of the Districts is *A*_3_≻*A*_2_≻*A*_1_≻*A*_4_,
3. when *q* ∈ [3.623,4.341], the ranking order of the Districts is *A*_3_≻*A*_2_≻*A*_4_≻*A*_1_,
4. when *q* ∈ [4.341,10.0], the ranking order of the Districts is *A*_3_≻*A*_4_≻*A*_2_≻*A*_1_.

**Figure 5:**
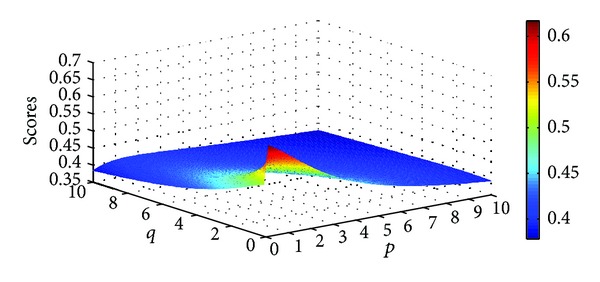
Scores for District *A*_1_ obtained by the IIFGWHM operator (*p* ∈ (0,6], *q* ∈ (0,6]).

**Figure 6:**
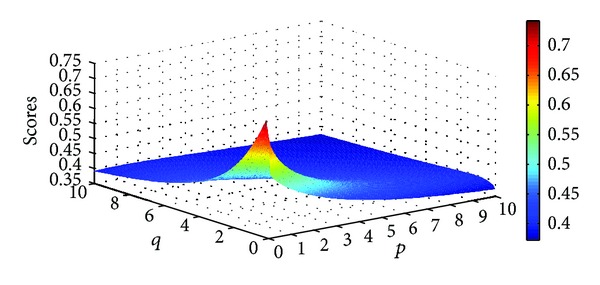
Scores for District *A*_2_ obtained by the IIFGWHM operator (*p* ∈ (0,6], *q* ∈ (0,6]).

**Figure 7:**
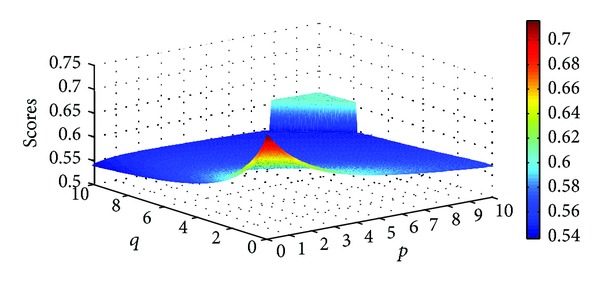
Scores for District *A*_3_ obtained by the IIFGWHM operator (*p* ∈ (0,6], *q* ∈ (0,6]).

**Figure 8:**
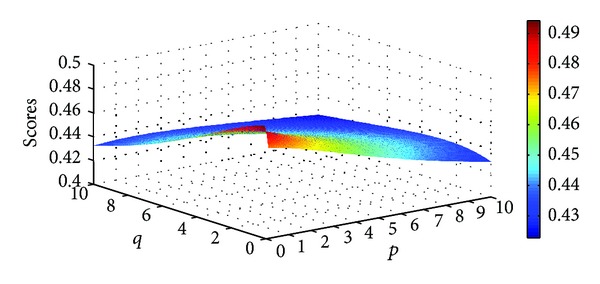
Scores for District *A*_4_ obtained by the IIFGWHM operator (*p* ∈ (0,6], *q* ∈ (0,6]).

Different scores of the four Districts can be obtained as the parameters *p* and *q* changed. Figures 5, 6, 7, and 8 illustrate the scores of four Districts obtained by the IIFGWHM operator in detail.

From the above analysis, we can easily find that when the parameters are assigned different values, different decision results may be generated. Therefore, it is a very flexible interval-valued intuitionistic fuzzy decision making method.

In order to compare the IIFGWHM operator with the IIFWBM operator which was proposed by Xu and Chen [33], we utilize the IIFWBM operator to replace (13) and analyze the decision making method. The IIFWBM operator was given as follows [33]:

(14) IIFWBM(α~1,α~2,…,α~n)=([(1−∏i=1,j=1i≠jn(1−(1−(1−ai)wi)p×(1−(1−aj)wj)q)1/n(n−1))1/(p+q), (1−∏i=1,j=1i≠jn(1−(1−(1−bi)wi)p×(1−(1−bj)wj)q)1/n(n−1))1/(p+q)], [1−(1−∏i=1,j=1i≠jn(1−(1−ciwi)p×(1−cjwj)q)1/n(n−1))1/(p+q),1−(1−∏i=1,j=1i≠jn(1−(1−diwi)p×(1−djwj)q)1/n(n−1))1/(p+q)]).

If we aggregate the IIFNs based on the IIFWBM operator, the aggregated IIFNs can be obtained as the values of the parameters *p* and *q* change. The results are shown in Table 5. The corresponding score values and the ranking of Districts are shown in Table 6.

**Figure 9:**
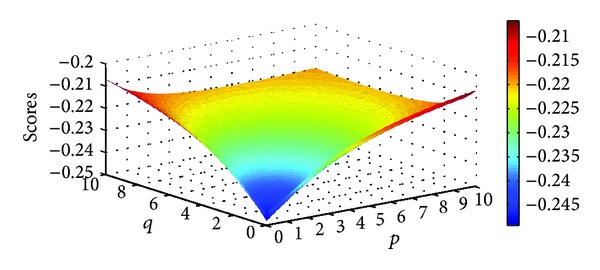
Scores for District *A*_1_ obtained by the IIFWBM operator (*p* ∈ (0,10], *q* ∈ (0,10]).

**Figure 10:**
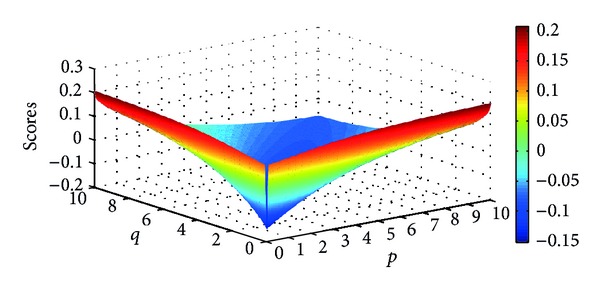
Scores for District *A*_2_ obtained by the IIFWBM operator (*p* ∈ (0,10], *q* ∈ (0,10]).

**Figure 11:**
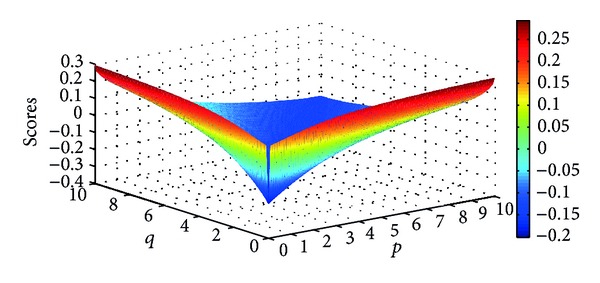
Scores for District *A*_3_ obtained by the IIFWBM operator (*p* ∈ (0,10], *q* ∈ (0,10]).

**Figure 12:**
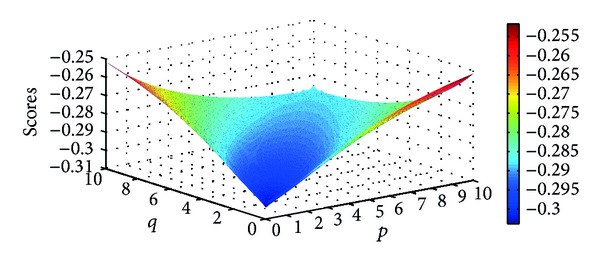
Scores for District *A*_4_ obtained by the IIFWBM operator (*p* ∈ (0,10], *q* ∈ (0,10]).

Different scores of the four Districts can be obtained as the parameters *p* and *q* change. Figures 9, 10, 11, and 12 illustrate the scores of four Districts obtained by the IFWBM operator in detail.

## 4. Concluding Remarks

In this paper, we have put forward an associated aggregation operator for IIFNs called the IIFGHM operator. We have analyzed the weighted form of IIFGHM operator and introduced the IIFGWHM operator. A flexible multicriteria decision making method has been introduced, by which the optimal alternative(s) can be derived. We have studied the applicability of the IIFGWHM operator in multicriteria decision making problems and we have carried out the evaluation of the performance of E-Government in Hangzhou city, China. In future research, we will consider other applications of this approach, such as investment management, teaching quality evaluation, and supply chain management.

## Figures and Tables

**Table 1:** The interval-valued intuitionistic fuzzy decision matrix *B*.

	*C*_1_	*C*_2_	*C*_3_
*A*_1_	([0.2, 0.3], [0.6, 0.7])	([0.2, 0.4], [0.4, 0.5])	([0.1, 0.3], [0.3, 0.5])
*A*_2_	([0.3, 0.5], [0.1, 0.3])	([0.3, 0.4], [0.4, 0.6])	([0.7, 0.9], [0.0, 0.1])
*A*_3_	([0.4, 0.6], [0.3, 0.4])	([0.7, 0.9], [0.0, 0.1])	([0.2, 0.3], [0.4, 0.6])
*A*_4_	([0.4, 0.6], [0.2, 0.3])	([0.3, 0.4], [0.4, 0.5])	([0.2, 0.4], [0.4, 0.5])

**Table 2:** The interval-valued intuitionistic fuzzy decision matrix *B*.

	*C*_1_	*C*_2_	*C*_3_
*A*_1_	([0.6, 0.7], [0.2, 0.3])	([0.2, 0.4], [0.4, 0.5])	([0.1, 0.3], [0.3, 0.5])
*A*_2_	([0.1, 0.3], [0.3, 0.5])	([0.3, 0.4], [0.4, 0.6])	([0.7, 0.9], [0.0, 0.1])
*A*_3_	([0.3, 0.4], [0.4, 0.6])	([0.7, 0.9], [0.0, 0.1])	([0.2, 0.3], [0.4, 0.6])
*A*_4_	([0.2, 0.3], [0.4, 0.6])	([0.3, 0.4], [0.4, 0.5])	([0.2, 0.4], [0.4, 0.5])

**Table 3:** Aggregated IIFNs based on IIFGWHM operator.

	*A*_1_	*A*_2_	*A*_3_	*A*_4_
*p* = 1, *q* = 1	([0.6107, 0.7546], [0.1246, 0.1830])	([0.6569, 0.7705], [0.1098, 0.1944])	([0.7351, 0.8111], [0.0913, 0.1675])	([0.6226, 0.7181], [0.1570, 0.2222])
*p* = 1, *q* = 7	([0.5398, 0.7056], [0.1727, 0.2278])	([0.5950, 0.7004], [0.1752, 0.2892])	([0.6862, 0.7564], [0.1181, 0.2027])	([0.6140, 0.6985], [0.1790, 0.2389])
*p* = 6, *q* = 3	([0.5363, 0.7016], [0.1732, 0.2296])	([0.5848, 0.7069], [0.1718, 0.2824])	([0.7006, 0.7646], [0.1193, 0.2030])	([0.6151, 0.6997], [0.1771, 0.2378])
*p* = 10, *q* = 1	([0.5236, 0.6902], [0.1839, 0.2425])	([0.5710, 0.7007], [0.1779, 0.2944])	([0.6954, 0.7605], [0.1210, 0.2071])	([0.6093, 0.6898], [0.1861, 0.2459])
*p* = *q* = 10	([0.5140, 0.6836], [0.1916, 0.2497])	([0.5665, 0.6836], [0.1916, 0.3145])	([0.6762, 0.7450], [0.0000, 0.2117])	([0.6049, 0.6834], [0.1916, 0.2512])

**Table 4:** Score values obtained by the IIFGWHM operator and the rankings of Districts.

	*A*_1_	*A*_2_	*A*_3_	*A*_4_	Ranking
*p* = 1, *q* = 1	0.5288	0.5616	0.6437	0.4807	*A*_3_≻*A*_2_≻*A*_1_≻*A*_4_
*p* = 1, *q* = 7	0.4224	0.4155	0.5609	0.4473	*A*_3_≻*A*_4_≻*A*_1_≻*A*_2_
*p* = 6, *q* = 3	0.4176	0.4188	0.5715	0.4500	*A*_3_≻*A*_4_≻*A*_2_≻*A*_1_
*p* = 10, *q* = 1	0.3937	0.3997	0.5640	0.4335	*A*_3_≻*A*_4_≻*A*_2_≻*A*_1_
*p* = *q* = 10	0.3781	0.3720	0.6048	0.4228	*A*_3_≻*A*_4_≻*A*_1_≻*A*_2_

**Table 5:** Aggregated IIFNs based on IIFWBM operator.

	*A*_1_	*A*_2_	*A*_3_	*A*_4_
*p* = 1, *q* = 1	([0.1006, 0.1855], [0.6761, 0.7619])	([0.1347, 0.2312], [0.5041, 0.7337])	([0.1523, 0.2290], [0.5928, 0.7507])	([0.0839, 0.1392], [0.7424, 0.8139])
*p* = 1, *q* = 7	([0.1636, 0.2286], [0.6689, 0.7444])	([0.2107, 0.3510], [0.2351, 0.6386])	([0.2985, 0.4560], [0.2717, 0.5350])	([0.1172, 0.1695], [0.7129, 0.7777])
*p* = 6, *q* = 3	([0.1388, 0.2171], [0.6718, 0.7493])	([0.1890, 0.2966], [0.3952, 0.6917])	([0.2147, 0.3227], [0.4655, 0.6632])	([0.0982, 0.1543], [0.7297, 0.7963])
*p* = 10, *q* = 1	([0.1770, 0.2391], [0.6658, 0.7395])	([0.2267, 0.3783], [0.1772, 0.6136])	([0.3287, 0.5029], [0.2059, 0.4899])	([0.1256, 0.1790], [0.7005, 0.7658])
*p* = *q* = 10	([0.0000, 0.2282], [0.6673, 0.7392])	([0.1939, 0.2947], [0.4418, 0.7035])	([0.1871, 0.2751], [0.5412, 0.7157])	([0.0000, 0.1593], [0.7309, 0.7951])

**Table 6:** Score values obtained by the IIFWBM operator and the rankings of Districts.

	*A*_1_	*A*_2_	*A*_3_	*A*_4_	Ranking
*p* = 1, *q* = 1	−0.2453	−0.1364	−0.1819	−0.3016	*A*_2_≻*A*_3_≻*A*_1_≻*A*_4_
*p* = 1, *q* = 7	−0.2201	0.0579	0.0922	−0.2717	*A*_2_≻*A*_3_≻*A*_1_≻*A*_4_
*p* = 6, *q* = 3	−0.2273	−0.0493	−0.0714	−0.2877	*A*_2_≻*A*_3_≻*A*_1_≻*A*_4_
*p* = 10, *q* = 1	−0.2133	0.1006	0.1485	−0.2608	*A*_3_≻*A*_2_≻*A*_1_≻*A*_4_
*p* = *q* = 10	−0.2196	−0.0735	−0.1331	−0.2858	*A*_2_≻*A*_3_≻*A*_1_≻*A*_4_
